# The McCormick Science Institute: Mission, Funding Guidelines, Governance, and 20 Years of Contributions to the Research and Public Health Communities Pertaining to Spices and Herbs

**DOI:** 10.1093/nutrit/nuaf185

**Published:** 2026-05-26

**Authors:** Hamed Faridi, Guy H Johnson

**Affiliations:** The McCormick Science Institute, Minneapolis, MN 55416, United States; The McCormick Science Institute, Minneapolis, MN 55416, United States

**Keywords:** McCormick Science Institute, spices, herbs

## Abstract

The McCormick Science Institute (MSI) is a research funding institute dedicated to advancing scientific understanding of the potential health benefits of culinary spices and herbs. The MSI is fully funded by McCormick and Company, Inc. In this article we describe the rationale for establishing the MSI, the types of studies considered for funding, metrics on the contributions MSI-funded studies have made to the literature, governance of the MSI, and funding guidelines. Additional information and all MSI-funded peer-reviewed studies are available at the MSI website www.mccormickscienceinstitute.com.

Abundant anecdotal and observational evidence has documented the historical use of herbs and spices for their health benefits in many civilizations.^1^ Herbs and spices have also been used to mask unpleasant tastes and odors of food, and later, to keep food fresh.^2^ From the dawn of biblical times, spices were prized for a wide variety of uses, including religious offerings, burial rituals, medicines, trade, and seasoning. The King of France and Emperor of the West, Charlemagne (ad 742-814), played an important role in developing and growing local herbs. He was the first leader to have farmers plant an abundance of culinary herbs such as anise, fennel, fenugreek, sage, thyme, parsley, and coriander.

Throughout the world, spices and herbs are frequently used in cuisine to improve flavor and to provide new tastes. The information age and social media have ushered in a new era of global cuisine sharing. Curious home cooks increasingly prepare meals from a wide range of ethnic heritages using an increasing array of spices. The United States Department of Agriculture (USDA) has reported that the consumption of spices in the United States has increased exponentially over the course of the previous half-century, and spices such as ginger, cinnamon, turmeric, and chili pepper are being used more frequently than ever before.^3^

There has also been a renewed interest recently in the health benefits of spices and herbs. The 2020-2025 USDA-HHS *Dietary Guidelines for Americans* states that “Spices and herbs can help flavor foods when reducing added sugars, saturated fat, and sodium, and they also can add to the enjoyment of nutrient-dense foods, dishes, and meals that reflect specific cultures.”^4^

One of the most exciting developments for spices in modern times is that scientific evidence (including the research sponsored by the McCormick Science Institute [MSI]) is accumulating that supports the anecdotal health benefits embraced by our ancestors. Culinary spices and herbs may have beneficial effects in areas such as heart health, cognition, and weight management as well as improving diet quality by making healthier foods more acceptable to consumers. An open-source compendium of such research funded by the MSI is available in the July/August 2023 supplement to *Nutrition Today* (https://journals.lww.com/nutritiontodayonline/toc/2014/09001). The body of evidence to support the wisdom of our ancestors throughout the ages continues to expand.

The MSI is a research Institute fully funded by the McCormick Company, Inc., that operates with complete autonomy from the business unit. It is a thought leadership gift of the McCormick Company for the benefit of everyone. The mission of the MSI is to increase scientific understanding of the potential health benefits of culinary spices and herbs by funding studies at leading academic institutions. In 2007, not a single United States university with ongoing research programs on the health effects of spices and herbs at culinary levels could be identified. Since then, more than 30 North American and European universities have conducted research sponsored by the MSI. The MSI is governed by a blue-ribbon Scientific Advisory Council (https://www.mccormickscienceinstitute.com/about/scientific-advisory-council) that provides guidance on the direction of future research. More than 100 peer-reviewed studies and monographs have been published since the inception of the MSI in 2006.

Studies funded by the MSI have examined the potential health benefits of spices and herbs in 3 broad categories: *Metabolic Health* (physiological effects of spices and herbs on outcome measures such as bioavailability, chronic disease risk factors, inflammatory markers, and the microbiome); *Diet Quality* (including the effects of spices and herbs on improving the taste, acceptability and/or consumption of healthier foods lower in added sugars, saturated fat, and/or sodium); and *Nutrition Education and Translation* (including the effects of educational messages regarding spices and herbs on knowledge, attitude, and behavior among consumers).

These studies have made noteworthy contributions to the literature and have been cited more than 10 000 times since 2017. In addition, such studies have been instrumental in supporting the *2020-2025 Dietary Guidelines for Americans* recommendation, “Spices and herbs can help flavor foods when reducing added sugars, saturated fat, and sodium, and they also can add to the enjoyment of nutrient-dense foods, dishes, and meals that reflect specific cultures.”

The MSI does not fund studies of proprietary products (including McCormick’s brand products) and has no interest in the rights to intellectual property. All studies funded by the MSI examine culinary amounts of spices and herbs as opposed to the unphysiological doses often used in dietary supplements. Only studies with healthy human participants or in vitro designs are considered. The MSI does not fund animal research or studies that use extracts or isolated bioactive compounds. The MSI does not lobby other organizations and has never advocated for a commercial brand. In addition, all MSI-funded studies are open access and available to anyone at the MSI website (https://www.mccormickscienceinstitute.com/our-research).

The MSI is pleased to present this summary of 20 years of sponsored research conducted mainly in the United States, but also in Canada, France, the Netherlands, and the United Kingdom. The reviews in this supplement are the outcome of more than 75 institutional review board–approved clinical trials conducted during the past 20 years in the aforementioned 3 broad areas of metabolic health, diet quality, and nutrition education/translation.

The articles included in this summary are freely available at the website of this journal (https://academic.oup.com/nutritionreviews?login=false) and at the MSI website (https://www.mccormickscienceinstitute.com/our-research).
